# Image analysis traits of multiple muscles and intermuscular/subcutaneous fat influence Japanese Black beef carcass price and genetic parameters

**DOI:** 10.5713/ab.23.0330

**Published:** 2024-01-20

**Authors:** Yuta Tamagawa, Mikiya Takahashi, Koichi Hagiya, Keigo Kuchida

**Affiliations:** 1 Obihiro University of Agriculture and Veterinary Medicine, Obihiro, Hokkaido 080-8555, Japan

**Keywords:** Carcass Unit Price, Fats, Heritability, Image Analysis, Muscles

## Abstract

**Objective:**

The purposes of this study were to investigate the relationship between carcass unit price per 1 kg (UP) and multiple muscles and intermuscular fat (IF)/subcutaneous fat of beef carcasses using image analysis of cross-section images for Wagyu beef cattle in Japan, and to estimate their genetic parameters.

**Methods:**

The carcasses used in this study were 1,807 Japanese Black (Wagyu) cattle (1,216 steers and 591 heifers). An analysis of variance was conducted with UP as the dependent variable and market date, age in months, sex, and image analysis traits (IAT) as fixed effects, and standard partial regression coefficients were calculated for each IAT on UP. Also, the heritability of each IAT that affected UP and genetic correlation among IAT vs carcass grading traits were estimated.

**Results:**

Not only IAT related to carcass grading traits, *M. trapezius dorsi*, *M. latissimus dorsi*, and IF traits were significant differences in UP (p<0.05). The heritability of IAT associated with UP was estimated at 0.38 to 0.85. The genetic correlations between the area and thickness of *M. trapezius dorsi* and *M. latissimus dorsi* vs rib eye area (REA) were estimated to be moderately positive (0.53 to 0.66), while the genetic correlations between the IF area percentage vs carcass weight, REA, and yield score were estimated to be negative (−0.40, −0.56, and −0.34).

**Conclusion:**

UP was influenced by various traits, including *M. trapezius dorsi*, *M. latissimus dorsi*, and IF traits, as well as image analysis associated with carcass grading traits. Since these IAT associated with UP had hereditary and desirable genetic correlations with carcass grading traits, these traits were also important for genetic improvement.

## INTRODUCTION

In Japan, beef carcasses are traded by auction and negotiation. An auction is a transaction between a seller and several buyers. After an auction, the carcass unit price per 1 kg (UP) determined by the auction is announced by the media. The UP may fluctuate due to factors such as seasonal demand conditions. On the other hand, negotiation is a direct transaction between the seller and the buyer. In this situation, the prices are determined based on the published auction price and the grading grade of the carcasses. Carcass grading is evaluated by the Japan Meat Grading Association (JMGA) graders. Meat grading is divided into meat quality grade (MQ) and yield grade. MQ is based on a five-level evaluation of marbling, meat color, meat firmness/texture, and fat color/quality for *M. longissimus dorsi*, *M. semispinalis capitis*, and *M. semispinalis dorsi*. MQ5 is the highest rating and MQ1 is the lowest. The yield grade is divided into three levels, A to C, calculated according to rib eye area (REA), rib thickness (RT), carcass weight (CWT), and subcutaneous fat thickness (SFT). A is the highest rating and C is the lowest.

Yamaki et al [1] and Hirooka and Matsumoto [2] reported that the degree of marbling significantly affected UP in carcass grading evaluation. Kim et al [3] reported a high genetic correlation between UP and marbling score. However, Iwasaki et al [4] reported that the impact of beef marbling standard (BMS) on price was declining. In addition, even if the carcass was traded at the same meat market on the same day and evaluated the same carcass grading, UP was very different. For example, the difference between the highest and lowest A4 steers UP on one day in the Hokkaido carcass market was 550 yen/kg [5]. If the carcass weighted 500 kg, the difference would be estimated at 280,000 yen. Therefore, it was shown that the UP was not significantly affected only by the carcass grading evaluation such as BMS, but also by the condition of the cross-section between the 6th and 7th ribs of the beef carcass.

Garrett and Hinman [6] reported higher fat content in the infraspinatus, *serratus ventralis*, *longissimus*, *gluteus medius*, *semimembranosus*, and *adductor muscles* increased the marbling score. Brackebusch et al [7] reported that marbling in the *longissimus* enables the prediction of the fat content of 15 muscles, including *adductor muscles* and *biceps femoris*. However, these reports have been based on studies using muscle separated from the carcass. Except for Japan, only a few known studies on the muscle obtained from carcass cross-sectional images exist.

On the other hand, in Japan, Kuchida et al [8,9] reported that when the degree of marbling in rib eye was similar, characteristics other than carcass grading, such as coarseness of marbling particles and rib eye shape, were considered in determining the UP. Takeo et al [10] reported that the new fineness index, which evaluates the degree of fine marbling in the rib eye, affected the UP. However, these reports were on the image analysis traits (IAT) of the *M. longissimus dorsi*. The relationship among other muscles around the *M. longissimus dorsi* or intermuscular fat (IF)/subcutaneous fat (SF) and UP, has yet to be widely studied.

Therefore, this study aimed to investigate the relationship between UP and multiple muscle and IF/SF traits calculated by image analysis of carcass cross-sectional images. Also, when traits were found to be related to UP, the heritability of these traits and their genetic relationship to carcass grading traits were examined.

## MATERIALS AND METHODS

### Carcass data

The data and samples used in the present study were obtained from the carcass records (Animal Care and Use Committee approval was not required). The carcasses used in this study were Japanese Black cattle shipped to a meat processing plant from January to December 2019 in Hokkaido, Japan. Among them, MQ1, those with carcass defects, those over 40 months of age, and those that won the carcass competition were excluded because their UP was over- or under-valued or animals were extremely few. After exclusion, 1,807 animals (1,216 steers and 591 heifers) were used in the analysis. The average UP change in the meat processing plants examined in this study showed strong correlations with those of other major meat processing facilities in Tokyo, Osaka, and Fukuoka, Japan, with respective correlation coefficients of 0.94, 0.93, and 0.96 (the transaction dates of the major meat processing facilities and the meat processing facilities in this study differ by about less than 5 days). Images of the cross-section between the 6th and 7th rib on the left side of the carcasses were taken using a mirror-type camera (HK-333; Hayasaka Ricoh Co., Ltd., Sapporo, Japan) during the carcass grading. JMGA graders graded the carcasses under the beef carcass grading standards.

### Image analysis traits

**Figure 1:**
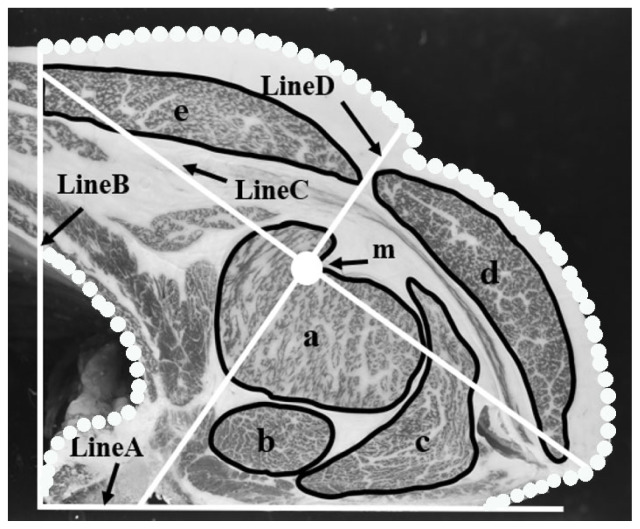
Region of interest of the measurements by image analysis at the cross-section between the 6th and 7th rib-bones. (a) *M. longissimus dorsi*; (b) *M. semispinalis capitis*; (c) *M. semispinalis dorsi*; (d) *M. trapezius dorsi*; (e) *M. latissimus dorsi*. LineA, line from the thoracic vertebra; LineB, vertical line to the thoracic vertebra; LineC, the first inertia principal axis passing through the center of gravity target field; LineD, carcass thickness; m, center of gravity of the target field.

Figure 1 shows the region of interest of the measurements by image analysis at the cross-section between the 6th and 7th rib. Five muscles extracted for image analysis were: *M. longissimus dorsi* (a), *M. semispinalis capitis* (b), *M. semispinalis dorsi* (c), *M. trapezius dorsi* (d), and *M. latissimus dorsi* (e). Nade et al [11] reported that the composition of a beef carcass can be accurately estimated by analyzing a cross-section carcass image of the area defined by the vertical line to the thoracic vertebra (Line A), the vertical line to the thoracic vertebra (Line B) and the dotted line in Figure 1. This area was used as the target field for this study. Line D shows the thickness of the carcass. This line is the second inertia principal axis which is orthogonal to the first inertia principal axis (line C), passing through the center of gravity (m) of the target field.

The IAT were calculated using beef carcass image analysis software (BeefAnalyzer-II Ver2.0; Meat Image Japan, Obihiro, Japan). Four traits were calculated for each of the target muscles: area (cm^2^), marbling percentage (%), coarseness index (%), and the new fineness index. Moreover, thickness was measured for *M. trapezius dorsi* and *M. latissimus dorsi*. Six traits were calculated for the target field analyzed: area (cm^2^), thickness (cm), IF area (cm^2^), IF area percentage (%), SF area (cm^2^), and SF area percentage (%).

The area of each muscle (cm^2^) was calculated by counting the number of pixels within each muscle and dividing by the number of pixels per cm^2^. The marbling percentage (%) is the area percentage of marbling particles within each muscle. The coarseness index (%) is an index of the degree of coarseness of marbling particles in each muscle [8]. The higher the coarseness index, the coarser the marbling particles contained in the muscle. The new fineness index was obtained by dividing the total circumference of marbling particles within each muscle by the square root of each muscle [12]. The higher the new fineness index, the finer the marbling contained. The thickness of each muscle (mm) is the length of an orthogonal line to the first inertia principal axis passing through the center of gravity of each muscle.

### Analysis of variance

To investigate the effect of IAT on UP, analysis of variance (ANOVA) was performed using the general linear model procedure in SAS (2019). The linear model used the equation according to Nomura and Kuchida [13], which was as follows:


$${UP}_{ijklmn}=D_{i}+A_{j}+{SEX}_{k}+F_{l}+{MQ}_{m}+{IAT}_{n}+{\left(MQ\times IAT\right)}_{mn}+e_{ijklmn}$$

Where *UP_ijklmn_* was the observation *ijklmn*th for UP, *D_i_* was the fixed effect of the ith carcass grading date (17 levels), *A_j_* was the fixed effect of the *j*th months of age (9 levels), *SEX_k_* was the fixed effect of the *k*th sex (2 levels), *F_l_* was the fixed effect of the *i*th fattening farm (166 levels), *MQ_m_* was the fixed effect of the *m*th MQ (4 levels), *IAT_n_* was the fixed effect of the *n*th IAT (28 levels) and *e_ijklmn_* was the residuals.

Furthermore, ANOVA was performed for each MQ, excluding the MQ from the fixed effects for models that interacted with the MQ and IAT in the model above. When ANOVA was conducted for each MQ, fattening farms were also excluded from the fixed effect because in MQ2, the number of heads was minimal (n = 50) and including fattening farms would make the analysis extremely unstable. The model was as follows:


$${UP}_{ijklm}=D_{i}+A_{j}+{SEX}_{k}+{IAT}_{l}+e_{ijkl}$$

### Genetic parameter estimation

Genetic parameters were estimated using the GIBBS1F90 program [14]. A single chain of 500,000 cycles was defined, with a burn-in of 100,000 cycles and a thinning interval of 10 cycles. Carcass grading traits, IAT and UP were analyzed. To examine the heritability of these traits, variance components and heritability were estimated using a one-trait animal model and genetic and phenotypic correlations were estimated using a two-trait animal model. Pedigree records were traced back to the 5th generation ancestors, and the number of animals was 10,381. The genetic parameters were estimated using the equation of Osawa et al [15], which is as follows:


$$Y_{ijklm}=H_{i}+T_{j}+{SEX}_{k}+A_{l}+u_{m}+e_{ijklm}$$

Where *Y_ijklm_* was the *ijklm*th observation for UP and IAT, *H_i_* was the fixed effect of the *i*th fattening farm (166 levels), *T_j_* was the fixed effect of the *j*th carcass grading season (4 levels), *SEX_k_* was the fixed effect of the *k*th sex (2 levels), *A_l_* was the fixed effect of the *l*th months of age (9 levels), *u_m_* was the random effect of the *m*th animal, and *e_ijklm_* was residual. The above model in matrix form is as follows:


y=Xβ+Za+e

where y is the vector of observation, β is the vector of fixed effects, a is the vector of random additive genetic effect and e is the vector of residual effect. The X and Z denote the incidence matrices relating y to β and a.

## RESULTS AND DISCUSSION

### Simple statistics

Table 1 shows simple statistics for carcass grading traits and IAT of Japanese Black cattle. Steers indicated higher IAT for most of each muscle than heifers. However, all SF traits (thickness, area, and area percentage) and IF area percentage were higher in heifers (28.9±8.1 mm, 88.4±23.7 cm^2^, 16.4%±3.3%, and 18.4%±2.5%) than in steers (23.0±6.8 mm, 75.7±20.9 cm^2^, 13.4%±3.0%, and 18.2%±2.3%). Similar to this study, Mueller et al [16] reported that SF thickness was thicker in heifers (16.50±0.60 mm) than in steers (11.99±0.59 mm) for Angus cattle. Sobczuk-Szul et al [17] also reported that heifers had greater fat attachment scores than steers for Holstein-Friesian×Limousin. Therefore, it was suggested that heifers had more IF and SF than steers.

The mean±standard deviation (SD) of UP was higher for steers (\ 2,261±264.0) than for heifers (¥ 2,180.8±254.5). The mean of BMS No. was higher for steers (7.5±2.3) than for heifers (6.8±2.2).

### Analysis of variance

Table 2 shows the F-value for each fixed effect in the ANOVA. MQ was significantly different in UP for all (p<0.01). This result agreed with the results of studies by Kim et al [3] and Ibi et al [18], who reported that marbling affects UP. Carcass grading date significantly differed in UP for all (p<0.01). In this study, the average UP for each carcass grading date differed by a maximum of 263.3 yen (max; 2,377.4 yen/kg, min; 2,114.0 yen/kg), suggesting that the carcass grading date significantly affected the UP. Significant differences were also observed for fattening farms, which is in line with the farm-specific effects on carcass prices reported by Gallo et al [19]. However, there were no significant differences in age in months. This finding contrasts with the results reported by Alam et al [20], who noted that the age in months at the time of slaughter impacted carcass prices in Holstein steers. We attribute this discrepancy to the fact that all animals in our study were approximately 30 months old, with no significant individual age variations. Many IAT showed significant interactions with MQ, but the new fineness index of *M. longissimus dorsi*, the marbling percentage and the new fineness index of *M. semispinalis capitis* and the new fineness index of *M. semispinalis dorsi* did not show interaction with MQ.

Table 3 shows the standardized partial regression coefficients for the UP on IAT that were shown to have significant interactions with MQ in Table 2. In MQ4 and MQ5, where the number of data was large enough, there was no difference in the results whether the fattening farm was included in the fixed effect. Therefore, the analysis was conducted using a formula that excludes the fattening farm. All muscle areas and thicknesses significantly differed in UP for all MQ and showed positive standardized partial regression coefficients. Those traits were highest (5.38 to 15.46) in MQ4. Thus, it was suggested that traits related to muscle size affect UP, and higher values of those traits have a positive effect on UP.

For the traits on marbling, the marbling percentage showed the highest standardized partial regression coefficient (6.85 to 10.31) in MQ5 for any muscle. This may be attributed to the fact that MQ5 has a broader range of marbling scores than the other MQ; range of marbling scores: MQ5, BMS8~12; MQ4, BMS5~7: MQ3, BMS3~4; MQ2, BMS2. The new fineness index was significant for *M. trapezius dorsi* and *M. latissimus dorsi* in UP at MQ4 and MQ5 and showed high standardized partial regression coefficients.

As for the target area, the area and thickness of the target area showed higher standard partial regression coefficients at lower MQ. Thus, these traits affect UP at lower MQ. IF and SF area and area percentage significantly differed in UP at MQ4 and MQ5 and showed negative standard partial regression coefficients (–3.18 to −9.98). Also, the area percentage was higher than the area for both fats. Sakoda et al [21] reported that carcasses with a larger area of “dice fat” which is part of the IF area, had a lower UP, consistent with this study. Therefore, UP increased with decreasing IF and SF area and area percentage at high MQ, suggesting that the effect was substantial for each fat area percentage.

### Genetic parameter estimation

In this study, genetic parameters were estimated focusing on the traits affecting UP, as shown in Table 3. In addition, since Takeo et al [10] reported that the new fineness index in *M. longissimus dorsi* affects UP, we also estimated genetic parameters for the new fineness index in *M. longissimus dorsi* not shown in Table 3. The heritability of UP was estimated to be 0.68 (Table 4). This is higher than the estimated 0.32 to 0.42 in Japanese Black cattle reported by Ibi et al [18] and the estimated 0.21 in Korean cattle reported by Kim et al [3]. In the UP vs carcass grading trait, moderate to high genetic correlations were estimated for REA, RT, yield score (YS), and BMS (0.78, 0.43, 0.78, and 0.98, respectively) (Table 5). However, no genetic correlation was found for UP vs CWT and SFT (0.10 and −0.05).

For the IAT related to carcass grading traits, the heritability of the area of *M. longissimus dorsi*, the marbling percentage of *M. longissimus dorsi* and *M. semispinalis dorsi* were estimated high (0.67, 0.85, and 0.66, respectively). Osawa et al [22] reported lower heritability of area and marbling percentage of *M. longissimus dorsi* (0.46 and 0.59) than this study. High positive genetic correlations were estimated for the area of *M. longissimus dorsi* vs REA, and the marbling percentage of *M. longissimus dorsi* and *M. semispinalis dorsi* vs BMS (1.00, 0.98 and 0.80, respectively). Osawa et al [22] reported genetic correlations between the area of *M. longissimus dorsi* vs REA and the marbling percentage of *M. longissimus dorsi* vs BMS (0.97 and 0.97), similar to this study. The marbling percentage of *M. longissimus dorsi* was estimated to have the highest genetic correlation with UP (0.97). Kim et al [3] reported a genetic correlation between the marbling score vs UP of 0.99, similar to this study. The heritability of the SF area and area percentage was estimated to be high (0.55 and 0.52). This was consistent with SF area in Japanese Black cattle (0.59) [23] and fat thickness (0.56 and 0.63) [24,25] in Hanwoo Cattle and Brahman cattle. A high positive genetic correlation with SF area and area percentage vs SFT was estimated (0.92 and 0.95). This suggests that measuring area is unnecessary in SF traits, and grading SFT is desirable.

In traits not included in carcass grading traits, heritability for the area and thickness of *M. trapezius dorsi* and *M. latissimus dorsi* was estimated to be high (0.55 to 0.63), and heritability for the new fineness index in *M. longissimus dorsi*, *M. trapezius dorsi*, and *M. latissimus dorsi* was also estimated to be high (0.69, 0.50, and 0.49, respectively). Osawa et al [23] reported higher heritability for the area of *M. trapezius dorsi* and *M. latissimus dorsi* (0.55 and 0.67), and Kato et al [26] reported higher heritability for the new fineness index of *M. longissimus dorsi* (0.62), which was similar to this study. The genetic correlations between the area and thickness of *M. trapezius dorsi* and *M. latissimus dorsi* vs REA were estimated to be moderately positive (0.52 to 0.66). The genetic correlations between the new fineness index of *M. longissimus dorsi*, *M. trapezius dorsi*, and *M. latissimus dorsi* vs BMS were estimated to be moderately positive (0.69, 0.53, and 0.64, respectively) and between those traits vs REA were estimated to be both moderately and highly positive (0.85, 0.60, and 0.58, respectively), and between those traits vs YS were estimated be moderately and highly positive (0.76, 0.58, and 0.53). Kato et al [26] reported genetic correlations between the new fineness index of *M. longissimus dorsi* vs BMS, REA, and YS were estimated to be moderately positive (0.69, 0.64, and 0.59), which were similar to or lower than this study. Therefore, since carcass grading traits such as BMS, REA, and YS also have a high genetic correlation with UP (Table 5), the new fineness index of each muscle was suggested to be a fundamental trait for the increase in UP.

Heritability for the IF area and area percentage was estimated to be 0.40 and 0.39. Osawa et al [23] reported heritability for the IF area (0.56 to 0.59), and this study was lower. The genetic correlations with IF area and area percentage vs BMS were estimated to be 0.17 and −0.09, respectively. Osawa et al [23] reported that the genetic correlation of IF area vs BMS was estimated to be positive (0.21 to 0.29), which differed from our study. The IF area vs SFT estimated a moderate positive genetic correlation (0.40), while the IF area percentage vs SFT estimated no genetic correlation (0.11). The IF area percentage vs UP was estimated to be −0.19, which is higher than the genetic correlations of SFT, SF area, and SF area percentage vs UP (–0.05, 0.06, and −0.09). Therefore, the IF area percentage is very different from the SF traits, suggesting that it may be a trait that was more effective than the SF traits for improving the UP.

Therefore, each IAT that affected UP had a hereditary and high genetic correlation with carcass grading traits, suggesting that they could be improved by improving them.

## CONCLUSION

The UP was suggested to be influenced by various traits, including *M. trapezius dorsi*, *M. latissimus dorsi*, and IF traits in addition to image analysis associated with carcass grading traits. Anderson et al [27] and Mendizabal et al [28] reported that image analysis allows marbling grading to be more accurate than human grading, and that beef carcass grading by image analysis has begun to be used in various countries around the world, including the United States and the European Union. This study obtained marbling traits and other traits simultaneously with image capture. Therefore, it is expected that new standards for carcass trading will be established based on the IAT obtained in this study, which will serve as the basis for auctions and negotiations. In addition, these IAT associated with UP had hereditary and desirable genetic correlations with carcass grading traits, suggesting that these traits were also important for breeding improvement. This would mean increased revenue for the farmer.

## Figures and Tables

**Table 1:** Simple statistics for carcass grading traits, image analysis traits and unit price (mean±standard deviation)

Traits	Steer (n = 1,216)	Heifer (n = 591)
Carcass grading traits		
CWT (kg)	491.9±54.2	452.8±53.4
REA (cm^2^)	62.7±11.0	59.7±9.7
RT (mm)	78.6±9.1	78.4±9.1
SFT (mm)	23.0±6.8	28.9±8.1
YS (%)	74.6±1.6	74.1±1.5
BMS	7.5±2.3	6.8±2.2
*M. longissimus dorsi*		
Area (cm^2^)	62.0±10.9	59.4±9.7
Marbling percentage (%)	51.8±7.7	50.0±7.6
New fineness index	89.9±12.1	88.2±11.6
Coarseness index (%)	15.7±4.9	15.9±5.0
*M. semispinalis capitis*		
Area (cm^2^)	14.6±2.8	11.7±2.8
Marbling percentage (%)	40.8±7.3	40.5±6.7
New fineness index	65.5±11.4	58.5±11.2
Coarseness index (%)	2.1±1.9	2.1±1.8
*M. semispinalis dorsi*		
Area (cm^2^)	39.3±6.7	36.5±5.5
Marbling percentage (%)	49.4±6.7	48.6±6.0
New fineness index	94.8±15.1	90.7±13.3
Coarseness index (%)	8.3±3.1	8.9±3.2
*M. trapezius dorsi*		
Area (cm^2^)	53.3±12.0	46.1±8.9
Marbling percentage (%)	44.4±7.7	36.8±7.1
Thickness (mm)	36.4±7.2	33.6±5.7
New fineness index	82.9±15.3	71.8±14.8
Coarseness index (%)	13.2±4.0	12.7±3.9
*M. latissimus dorsi*		
Area (cm^2^)	44.2±10.0	39.3±8.4
Marbling percentage (%)	46.0±8.3	40.2±8.0
Thickness (mm)	35.9±6.1	32.9±5.2
New fineness index	77.2±11.4	75.0±12.6
Coarseness index (%)	12.0±4.1	10.6±3.9
Target field		
Area (cm^2^)	560.0±64.0	533.9±62.7
Thickness (mm)	234.0±24.6	239.4±24.8
IF area (cm^2^)	102.1±16.8	98.2±16.9
IF area percentage (%)	18.2±2.3	18.4±2.5
SF area (cm^2^)	75.7±20.9	88.4±23.7
SF area percentage (%)	13.4±3.0	16.4±3.3
Price		
UP (yen)	2,261±264.0	2,180±254.5

CWT, carcass weight; REA, rib eye area; RT, rib thickness; SFT, SF thickness; YS, yeild score; BMS, beef marbling standard; IAT, image analysis traits; IF, intermuscular fat; SF, subcutaneous fat; UP, carcass unit price per 1 kg.

**Table 2:** F-values from analysis of variance of date, months of age, sex, farm, MQ, IAT, and MQ×IAT on UP

Traits	Date	Month of age	Sex	Farm	MQ	IAT	MQ×IAT
*M. longissimus dorsi*							
Area	27.2**	0.3	2.4	2.2**	41.0**	227.3**	5.8**
Marbling ratio	23.0**	0.4	4.9*	1.9**	13.7**	68.5**	14.2**
New fineness index	23.1**	0.5	5.0*	2.0**	11.8**	51.6**	0.4
Coarseness index	25.5**	0.8	7.4**	2.0**	104.5**	30.4**	3.4*
*M. semispinalis capitis*							
Area	21.6**	0.9	0.0	2.1**	67.8**	29.7**	2.9*
Marbling ratio	22.1**	0.7	6.9**	2.2**	14.8**	11.4**	1.6
New fineness index	22.0**	0.8	0.2	2.2**	14.7**	7.5**	2.3
Coarseness index	21.2**	0.6	5.5*	2.1**	453.2**	0.9	2.3
*M. semispinalis dorsi*							
Area	25.4**	0.5	0.8	2.1**	46.6**	83.7**	4.4**
Marbling ratio	23.2**	0.7	6.7*	2.0**	15.7**	63.9**	8.4**
New fineness index	24.8**	0.7	2.6	2.0**	16.1**	38.1**	1.0
Coarseness index	24.8**	0.7	5.9*	2.0**	166.7**	9.3**	4.3**
*M. trapezius dorsi*							
Area	24.1**	0.5	0.1	2.0**	61.1**	84.7**	5.1**
Marbling ratio	23.8**	0.7	0.1	2.1**	13.8**	13.4**	9.8**
Thickness	24.1**	0.4	1.2	2.1**	46.2**	79.3**	5.4**
New fineness index	21.9**	0.5	0.0	2.0**	17.4**	16.4**	3.4*
Coarseness index	24.3**	0.7	6.8**	2.1**	133.0**	13.6**	8.4**
*M. latissimus dorsi*							
Area	23.8**	0.7	0.5	2.1**	94.2**	132.9**	11.8**
Marbling ratio	23.3**	0.6	0.8	2.1**	20.1**	22.0**	17.1**
Thickness	24.0**	0.6	0.3	2.0**	41.0**	102.0**	9.1**
New fineness index	22.2**	0.5	5.7*	2.0**	23.7**	57.7**	4.0**
Coarseness index	24.9**	0.6	3.3	2.1**	141.2**	29.8**	9.7**
Target field							
Area	20.6**	0.5	2.5	2.3**	50.9**	91.0**	13.9**
Thickness	21.**	0.4	10.5**	2.3**	42.1**	75.7**	12.5**
IF area	21.1**	0.4	5.6*	2.2**	106.1**	21.3**	21.2**
IF area ratio	21.1**	0.5	3.9*	2.2**	63.9**	1.3	16.3**
SF area	22.1**	0.5	0.4	2.1**	147.0**	4.9*	17.5**
SF area ratio	22.2**	0.6	2.1	2.1**	92.5**	1.2	12.0**

UP, carcass unit price per 1 kg; IAT, image analysis traits; MQ, meat quality grade; IF, intermuscular fat; SF, subcutaneous fat.

* p<0.05;

** p<0.01.

**Table 3:** Standardized partial regression coefficient of image analysis traits on unit price from analysis of variance for each meat quality

IAT	Standardized partical regression coefficient			

	MQ2	MQ3	MQ4	MQ5

	n = 50	n = 269	n = 786	n = 702
*M. longissimus dorsi*				
Area	4.34**	9.12**	15.46**	11.31**
Marbling percentage	3.15**	1.59	6.38**	10.31**
Coarseness index	2.07*	1.91	3.48**	3.98**
*M. semispinalis capitis*				
Area	2.67*	3.28**	5.38**	2.62**
Coarseness index	0.36	1.44	1.11	−2.48*
*M. semispinalis dorsi*				
Area	4.18**	4.92**	8.94**	4.65**
Marbling percentage	2.29*	2.61**	4.98**	8.74**
Coarseness index	2.95**	1.18	−1.02	−0.43
*M. trapezius dorsi*				
Area	3.43**	3.80**	9.21**	6.77**
Marbling percentage	1.78	−0.73	3.33**	6.85**
Thickness	4.10**	3.17**	7.60**	6.47**
New fineness index	1.09	0.42	8.13**	6.92**
Coarseness index	4.16**	0.95	−2.28*	−0.31
*M. latissimus dorsi*				
Area	4.72**	6.23**	6.61**	6.18**
Marbling percentage	1.54	−0.77	3.11**	7.40**
Thickness	4.00**	4.91**	5.69**	7.84**
New fineness index	2.75*	2.14*	5.61**	7.00**
Coarseness index	3.29**	2.29*	−0.32	2.35*
Target field				
Area	6.24**	4.48**	3.59**	2.13*
Thickness	3.88**	2.87**	2.60**	1.31
IF area	5.58**	2.90**	−1.26	−3.18**
IF area percentage	3.96**	0.82	−4.84**	−5.28**
SF area	4.67**	−1.50	−6.81**	−4.34**
SF area percentage	2.59*	−3.79**	−9.98**	−5.88**

IAT, image analysis traits; MQ, meat quality grade; IF, intermuscular fat; SF, subcutaneous fat.

* p<0.05;

** p<0.01.

**Table 4:** Posterior means (standard deviation) in parentheses of heritability for image analysis traits and unit price

Traits	Heritability
*M. longissimus dorsi*	
Area	0.67 (0.13)
Marbling percentage	0.85 (0.10)
New fineness index	0.69 (0.13)
*M. semispinalis capitis*	
Area	0.38 (0.10)
*M. semispinalis dorsi*	
Area	0.46 (0.09)
Marbling percentage	0.66 (0.12)
*M. trapezius dorsi*	
Area	0.55 (0.12)
Marbling percentage	0.58 (0.12)
Thickness	0.58 (0.12)
New fineness index	0.50 (0.12)
*M. latissimus dorsi*	
Area	0.66 (0.11)
Marbling percentage	0.72 (0.11)
Thickness	0.63 (0.10)
New fineness index	0.49 (0.12)
Target field	
Area	0.62 (0.13)
Thickness	0.52 (0.13)
IF area	0.40 (0.11)
IF area percentage	0.39 (0.10)
SF area	0.55 (0.11)
SF area percentage	0.52 (0.10)
UP	0.68 (0.13)

IAT, image analysis traits; UP, carcass unit price per 1 kg; IF, intermuscular fat; SF, subcutaneous fat.

**Table 5:** Posterior means and standard deviation of genetic correlation estimates between carcass grading traits vs unit price

Traits	Genetic correlation	SD
CWT	0.11	0.17
REA	0.78	0.08
RT	0.43	0.14
SFT	−0.05	0.17
YS	0.78	0.08
BMS	0.98	0.01

SD, standard deviation; UP, carcass unit price per 1 kg; CWT, carcass weight; REA, rib eye area; RT, rib thickness; SFT, subcutaneous fat thickness; YS, yield score; BMS, beef marbling standard.

**Table 6:** Posterior means (standard deviation) of genetic correlation estimates between carcass grading traits and unit price vs image analysis traits

Traits	Carcass graiding traits and unit price						

	CWT	REA	RT	SFT	YS	BMS	UP
*M. longissimus dorsi*							
Area	0.44 (0.14)	1.00 (0.00)	0.42 (0.15)	−0.21 (0.18)	0.90 (0.04)	0.70 (0.09)	0.74 (0.09)
Marbling percentage	0.15 (0.15)	0.65 (0.09)	0.42 (0.14)	0.12 (0.17)	0.58 (0.10)	0.98 (0.01)	0.97 (0.01)
New fineness index	0.26 (0.16)	0.85 (0.05)	0.22 (0.17)	−0.18 (0.17)	0.76 (0.07)	0.69 (0.09)	0.75 (0.07)
*M. semispinalis capitis*							
Area (cm^2^)	0.52 (0.14)	0.43 (0.15)	0.40 (0.16)	−0.02 (0.18)	0.31 (0.16)	0.12 (0.18)	0.10 (0.19)
*M. semispinalis dorsi*							
Area	0.32 (0.16)	0.69 (0.10)	0.30 (0.17)	−0.14 (0.18)	0.63 (0.11)	0.37 (0.15)	0.51 (0.14)
Marbling percentage	−0.15 (0.18)	0.55 (0.12)	0.22 (0.14)	0.00 (0.17)	0.59 (0.12)	0.80 (0.07)	0.88 (0.05)
*M. trapezius dorsi*							
Area	0.32 (0.15)	0.66 (0.10)	0.44 (0.15)	0.00 (0.18)	0.58 (0.12)	0.40 (0.14)	0.53 (0.12)
Marbling percentage	0.12 (0.18)	0.57 (0.12)	0.43 (0.14)	0.29 (0.16)	0.48 (0.13)	0.68 (0.09)	0.66 (0.10)
Thickness	0.30 (0.16)	0.57 (0.12)	0.45 (0.14)	0.14 (0.17)	0.46 (0.13)	0.34 (0.14)	0.46 (0.14)
New fineness index	0.05 (0.20)	0.60 (0.12)	0.23 (0.19)	−0.07 (0.18)	0.58 (0.12)	0.53 (0.12)	0.58 (0.12)
*M. latissimus dorsi*							
Area	0.66 (0.09)	0.60 (0.10)	0.53 (0.12)	0.16 (0.16)	0.34 (0.14)	0.36 (0.13)	0.43 (0.13)
Marbling percentage	−0.04 (0.19)	0.48 (0.12)	0.16 (0.17)	0.18 (0.17)	0.42 (0.14)	0.67 (0.09)	0.74 (0.08)
Thickness	0.48 (0.12)	0.52 (0.13)	0.37 (0.15)	0.05 (0.18)	0.34 (0.14)	0.36 (0.13)	0.45 (0.13)
New fineness index	0.27 (0.19)	0.58 (0.14)	0.38 (0.18)	−0.02 (0.20)	0.53 (0.15)	0.64 (0.11)	0.67 (0.11)
Target field							
Area	0.84 (0.06)	0.62 (0.11)	0.83 (0.07)	0.45 (0.15)	0.34 (0.16)	0.33 (0.15)	0.48 (0.15)
Thickness	0.73 (0.11)	0.42 (0.17)	0.83 (0.08)	0.68 (0.12)	0.10 (0.21)	0.29 (0.18)	0.41 (0.18)
IF area	0.38 (0.16)	0.02 (0.20)	0.71 (0.11)	0.40 (0.16)	−0.07 (0.19)	0.17 (0.18)	0.19 (0.18)
IF area percentage	−0.40 (0.17)	−0.56 (0.15)	0.10 (0.18)	0.11 (0.17)	−0.33 (0.17)	−0.09 (0.17)	−0.19 (0.18)
SF area	0.58 (0.11)	−0.02 (0.17)	0.61 (0.12)	0.92 (0.03)	−0.39 (0.15)	0.04 (0.16)	0.06 (0.17)
SF area percentage	0.31 (0.16)	−0.32 (0.17)	0.41 (0.15)	0.95 (0.03)	−0.54 (0.27)	−0.09 (0.16)	−0.09 (0.17)

IAT, image analysis traits; UP, carcass unit price per 1 kg; CWT, carcass weight; REA, rib eye area; RT, rib thickness; SFT, subcutaneous fat thickness; YS, yield score; BMS, beef marbling standard; IF, intermuscular fat; SF, subcutaneous fat.

## References

[1] Yamaki H, Hasegawa T, Ito H, Ugiie T (1996). Beef carcass grading pricing factors for buyers. Collected papers of Japan Economic Review.

[2] Hirooka H, Matsumoto M (1998). Factors involved in price determination in the Japanese beef carcass market. Agric Econ Res.

[3] Kim J, Kim D, Lee J, Lee C (2010). Genetic relationship between carcass traits and carcass price of Korean cattle. Asian-Australas J Anim Sci.

[4] Iwasaki R, Sakoda K, Asa R, Goto T, Hagiya K, Kuchida K (2017). Evaluation of overall sire bulls using image analysis traits in the Japanese Black cattle. Nihon Chikusan Gakkaiho (Anim Sci J?).

[5] Hokkaido Dairy and Livestock Association (c2013). Hokuren Tokachi carcass market results from each standard [Internet].

[6] Garrett WN, Hinman N (1971). Fat content of trimmed beef muscles as influenced by quality grade, yield grade, marbling score and sex. J Anim Sci.

[7] Brackebusch SA, Mckeith FK, Carr TR, Mclaren DG (1991). Relationship between longissimus composition and the composition of other major muscles of the beef carcass. J Anim Sci.

[8] Kuchida K, Suzuki M, Miyoshi S (2001). Evaluation of coarseness of marbling in the beef rib eye by computer image analysis. Anim Sci J.

[9] Kuchida K, Kikuchi A, Kato K, Hidaka S, Suzuki M, Miyoshi S (2003). Evaluation method for the shape of M. longissimus thoracis by computer image analysis and effect of sire on the shape in Japanese Black. Anim Sci J.

[10] Takeo A, Asa R, Hagiya K, Kuchida K (2016). Effect of marbling shape on unit price in the M. longissimus dorsi of Japanese Black and crossbred breeds. Anim Sci J.

[11] Nade T, Karnuah AB, Masuda Y, Hirabara S, Fujita K (2001). Estimation of carcass composition from the cross-section at ribloin of Japanese Black steers by computer image analysis. Anim Sci J.

[12] Kuchida K, Kanai T, Obihiro University of Agriculture and Veterinary Medicine, Japan Meat Grading Association Methods for evaluating marbling of meat [2012 Sep 28]. Japanese patent.

[13] Nomura N, Kuchida K (2022). Effects of monounsaturated fatty acid compositions of intermuscular fat on carcass price of Japanese Black cattle. Anim Sci J.

[14] Misztal I, Tsuruta S, Strabel T, Auvray B, Druet T, Lee DH BLUPF90 and related programs (BGF90).

[15] Osawa T, Kuchida K, Kato T, Suzuki M, Miyoshi S (2004). Estimation of genetic parameters for image analysis traits of carcass cross-section and carcass traits in Japanese Black cattle. Anim Sci J.

[16] Mueller LF, Balieiro JCC, Ferrinho AM (2019). Gender status effect on carcass and meat quality traits of feedlot Angus × Nellore cattle. Anim Sci J.

[17] Sobczuk-Szul M, Mochol M, Nogalski Z, Pogorzelska-Przybyłek P (2021). Fatty acid profile as affected by fat depot and the sex category of Polish Holstein-Friesian×Limousin fattening cattle fed silage ad libitum. Anim Sci J.

[18] Ibi T, Kahi AK, Hirooka H (2006). Effect of carcass price fluctuations on genetic and economic evaluation of carcass grading traits in Japanese Black cattle. J Anim Sci.

[19] Gallo L, Sturaro E, Bittante G (2017). Body traits, carcass characteristics and price of cull cows as affected by farm type, breed, age and calving to culling interval. Animal.

[20] Alam M, Cho KH, Lee SS (2013). Effect of carcass traits on carcass prices of Holstein steers in Korea. Asian-Australas J Anim Sci.

[21] Sakoda K, Maeda S, Asa R, Hagiya K, Kuchida K (2016). Effects of “Dice fat” area on carcass grading traits, image analysis traits, and UP in Japanese Black cattle. Anim Sci J.

[22] Osawa T, Kuchida K, Hidaka S, Kato T (2008). Genetic parameters for image analysis traits on M. longissimus thoracis and M. trapezius of carcass cross section in Japanese Black steers. J Anim Sci.

[23] Osawa T, Hasegawa M, Kuchida K, Hidaka S, Sekikawa M, Tsukuda H (2004). Estimation of genetic parameters for muscle area, subcutaneous fat of carcass cross-section in Japanese Black cattle. Anim Sci J.

[24] Naserkheil M, Lee DH, Kong HS, Seong J, Mehrban H (2021). Estimation of genetic parameters and correlation between yearling ultrasound measurements and carcass traits in Hanwoo cattle. Animals.

[25] Riley DG, Chase CCJ, Hammond AC (2002). Estimated genetic parameters for carcass traits of Brahman cattle. J Anim Sci.

[26] Kato K, Maeda S, Kuchida K (2014). Genetic parameters for fineness of marbling in M. longissimus thoracis of Japanese Black cattle. Anim Sci J.

[27] Anderson F, Cook J, Williams A, Gardner GE (2018). Computed tomography has improved precision for prediction of intramuscular fat percent in the M. longissimus thoracis et lumborum in cattle compared to manual grading. Meat Sci.

[28] Mendizabal JA, Ripoll G, Urrutia O, Insausti K, Soret B, Arana A (2021). Predicting beef carcass fatness using an image analysis system. Animals.

